# Supplementary material for: Chromothripsis during telomere crisis is independent of NHEJ, and consistent with a replicative origin
Source: Genome Res. 2019 May;29(5):737–49. doi: 10.1101/gr.240705.118 (PMC6499312; doi:10.1101/gr.240705.118)
Supplement: Supplemental Material [file supp_gr.240705.118_Supplemental_file_1.zip › contigs/annotated_contigs/DB46/contig.2.DB46_length_502_mean_cov_5.61354581673.docx]

**DB46_length_502_mean_cov_5.61354581673**

```
TGGCTTTTGGGAGCAGGTGGCTTTTGGTTACATGGAAAAGCTCTTCAGTGGCGATTTCTAAGATTTTGGTGAACCCGTCACTTGAGCAG
 >chr14:86627636-86627857 - E=2e-121
TGTACATTGTACCCAGCGTGTAGTCTTTTATCCCTCACTTCCCTCCCAAACTTCCTCTCAATTCCCCAAAATCCATCATATCATTCTTA

TGCCTTTGTGTCCTCATAGCTTAGGCCCCACTTATAAG|GGAAA|CAGCAAAACAACTCATTAACATTTTAATAATCTATGTTTTAATG
                                       >chr14:86607036-86607287 + E=2e-109
AAACCTGCAAAGGTAAGAGGAATATGCACATGTGGTCATATAAATGAAAAATACAAAAACCAGTAGAGTTATTATATTTTCTTACTGCC

AGGGCTGCCTTGCAGCCAGGGTAGCTAAGATAATGTCCATCTTGAGAACCCAAGGTCTTTTTTTTTTTTTTTTTTTTCATGTAAATATT

TACTGTTATCATTTTTGTATTCTT|TTACGATCCTGTTTTTTTTTTGTATCTAATTTTAT
```
